# Common Variable Immunodeficiency and Peripheral Lung Nodule

**DOI:** 10.4137/ccrep.s735

**Published:** 2008-06-20

**Authors:** Miguel Ángel Núñez, Javier Velasco, Jose Luis Hernandez, Cristina Castrillo, Maria Luisa Cagigal

**Affiliations:** 1 Department of Internal Medicine and Pathology. Hospital Marqués de Valdecilla. University of Cantabria, 39008 Santander, Spain.

## Introduction

Cryptococcus neoformans is an encapsulated yeast first isolated by Busse in 1894 from the tibia of a 31-year-old woman [1]. Lungs are considered the initial site of almost all cryptoccocal infections, and the second clinically more relevant after central nervous system. The pattern of pulmonary disease is extremely variable, ranging from asymptomatic saprophytic airway colonization to acute respiratory distress syndrome, mainly observed in immunocompromised patients [2].

Exposition to Cryptococcus is virtually universal, and usually occurs during childhood. It is thought that humans become infected after inhalation of the fungus. Afterwards, it causes a little focal pneumonitis which may or may not becomes symptomatic, mainly depending on the immune status of the host. In this initial phase of infection, animal models have demonstrated that alveolar macrophages, natural killer cells and helper T-cells are the most important host defence mechanisms against the fungus. In fact, the most serious infections usually occur in subjects with conditions associated with defective cell-mediated immunity, such as acquired immunodeficiency syndrome (AIDS), cirrhosis, renal failure, sarcoidosis, chronic lung disease, diabetes, stem-cell and solid organ transplantation, malignancies, and treatment with long-term corticosteroids of tumour necrosis factor (TNF) antagonists. However, patients with immunoglobulin deficiency are less prone to develop this fungal infection [3].

## Case Report

A 28-year-old man, presented with a history of pleuritic right chest pain and haemoptysis for 2 days. The pain was intermittent and worse when he lies down and improved in standing position. It did not radiate, and was no reproducible with palpation. He also noticed a small amount of blood in the sputum but denied any traumatic injury, and he did not have other symptoms such as dyspnoea, fever, chills, night sweats of weight loss.

Six years earlier a sclerosing mesenteritis with retroperitoneal lymphadenopathy, hypoplasia of the inferior vena cava, and thrombosis with cavernomatous transformation of the portal vein have been diagnosed, and he was treated with prednisone and acenocoumarol. Moreover, a diagnosis of common variable immunodeficiency had been made four years before, and the patient was started on monthly regimen of intravenous immunoglobulin.

He was an ex-smoker of 15 pack-years since one month before admission, and consumed 2–3 alcoholic drinks on occasional weekends. His current medications included prednisone (10 mg/day), colchicine, enalapril, weekly risedronate, and acenocoumarol. He had neither recent travel history, nor noticed environmental exposures, and his family history was unremarkable.

The patient was in good health status with normal body weight and had normal vital signs. Chest examination showed no abnormalities. Neither lymphadenopathy nor organomegaly were detected. The reminder of physical examination was unremarkable except for the presence of collateral circulation in the anterior abdominal wall and both legs.

**Figure 1:**
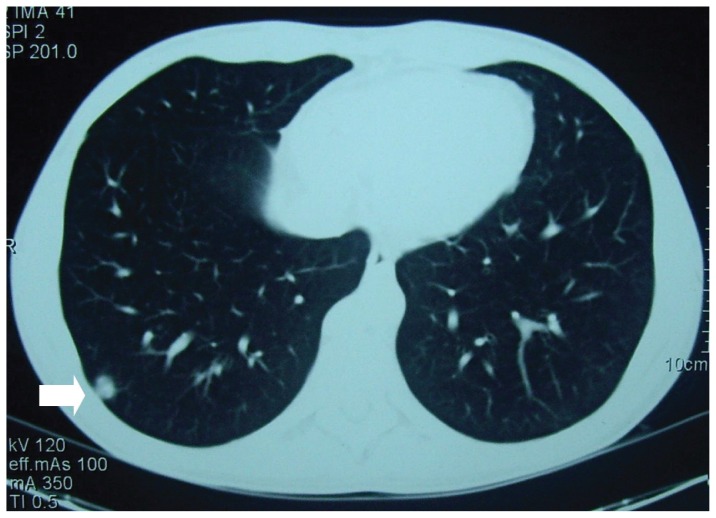
CT-scan of the chest showing a well-circumscribed mass in the right lung basis (arrow).

**Figure 2A and 2B:**
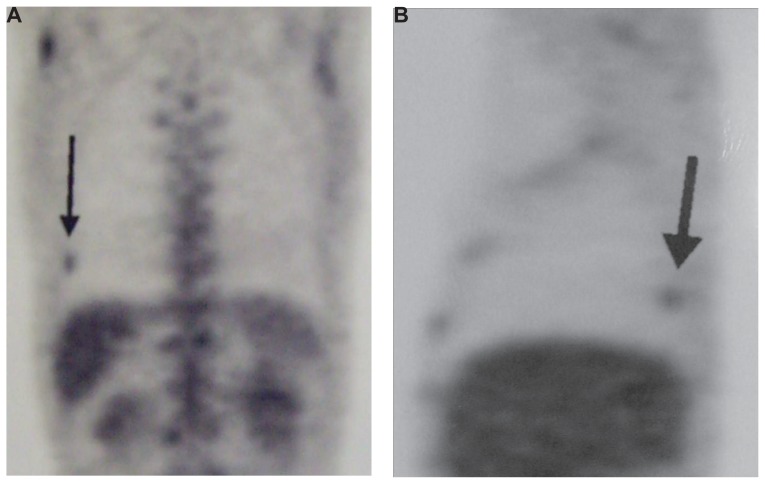
PET scan demonstrating a focus of intense FDG activity (arrows) in the right lung basis.

**Figure 3:**
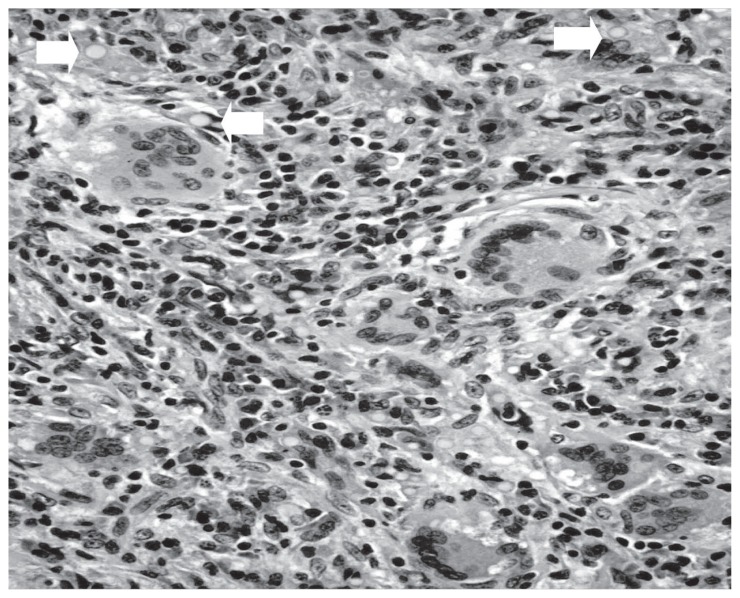
Inflammatory infiltrate with lymphocytes, macrophages, giant cells and numerous round cryptoccoci (arrows).

Hemogram, blood chemistry profile and urinalysis were within normal limits. The international normalized ratio was 1.97. Sputum and blood cultures were sterile. He tested negative for human immunodeficiency virus (HIV). An electrocardiogram showed a normal sinus rhythm. As plain chest radiography showed no abnormalities, an iodine-based contrast enhanced thoracic multidetector computed tomography (CT) was performed. A rounded peripheral well-defined nodule, 1.23 cm in diameter, was detected in the right lung basis (Fig. 1). There was no evidence of lymphadenopathy. To rule out malignancy he underwent a 18-fluorodesoxiglucose positron emission tomography (FDG-PET) scan which showed intense focal uptake in the nodule with a degree of activity highly suspicious of neoplastic origin (Fig. 2A and 2B). There were no other metabolically active lesions in the lungs. Although a fine needle aspiration biopsy was considered, it was thought prudent to confirm the diagnosis with complete excision of the nodule by video-assisted thoracoscopy. The procedure was performed without complications and resection specimen was collected and sent for pathologic analysis (Fig. 3) and a diagnosis of pulmonary cryptococcosis was made.

## Discussion

The presentation features in HIV-negative immunocompromised patients with pulmonary cryptococcosis have been recently reviewed in 109 patients [4]. The most common symptoms were cough (61%), dyspnoea (48%), fever (29%), weight loss (19%), pleuritic chest pain (19%), and night sweats (16%).

From a radiological point of view, features of pulmonary cryptococcosis vary widely. The most frequent radiographic findings are peripheral well-defined, non-calcified and non-cavitated nodules. Lobar infiltrates, hilar and mediastinal lymphadenopathy, and pleural effusions can occur to a lesser extent [5].

Most of infections developing in immunocompromised individuals, as our patient, are thought to be secondary to latent infection reactivation, and this group of subjects is more prone to suffer disseminated disease. Our patient was on corticosteroids during the last six years to control its sclerosing mesenteritis. This fact probably put him at high risk to develop cryptoccocal infection. The role that common variable immunodeficiency has played in the pathogenesis of the fungal disease remains speculative, since this case represents, to our knowledge, the first reported one of such association [6].

On the other hand, another interesting feature of our case is the value of FDG-PET for the diagnosis of pulmonary nodules. As a matter of fact, FDG-PET has come to play an increasingly important role to differentiate malignant from benign lesions and to stage and follow patients with lung neoplasms [7]. However, although PET has demonstrated sensivity and specificity rates of 80%–90% for the diagnosis of malignancies, is a procedure that also gives false positive and negative results [8]. In our patient, FDG-PET hypercaptation was suspicious of a malignant lesion, considering that patients with common variable immunodeficiency are at increased risk of developing solid or hematological neoplasms, such a non-Hodgkin’s lymphoma.

Our patient was treated with IV liposomal amphotericin B (5 mg/Kg/day) for three weeks. Cryptococcal antigen was negative, and a cranial magnetic resonance imaging (MRI) showed no evidence of central nervous system dissemination. A thoracic CT-scan performed four weeks later did not showed any pulmonary lesion. He completed a 3-month course of oral fluconazole (400 mg/day). Six months after discharge the patient felt well without any evidence of recurrence. He recalled to have been cleaning pigeon excrements at the window of his room several weeks before admission.

In conclusion, pulmonary cryptococcosis must be born in mind in the differential diagnosis of a solitary lung nodule, especially in immunocompromised hosts or in patients on long-term corticosteroid therapy. Moreover, our case emphasizes the limitations of specificity of FDG-PET in characterizing pulmonary nodules, even when complemented by other imaging techniques such as CT-scan, and the importance of prompt rule out or confirm suspected malignancy by cytological diagnosis.
